# Differences in Biofilm Mass, Expression of Biofilm-Associated Genes, and Resistance to Desiccation between Epidemic and Sporadic Clones of Carbapenem-Resistant *Acinetobacter baumannii* Sequence Type 191

**DOI:** 10.1371/journal.pone.0162576

**Published:** 2016-09-13

**Authors:** Gati Noble Selasi, Asiimwe Nicholas, Hyejin Jeon, Seok Hyeon Na, Hyo Il Kwon, Yoo Jeong Kim, Sang Taek Heo, Man Hwan Oh, Je Chul Lee

**Affiliations:** 1 Department of Microbiology, Kyungpook National University School of Medicine, Daegu, Republic of Korea; 2 Department of Internal Medicine, Jeju National University School of Medicine, Jeju, Republic of Korea; 3 Department of Nanobiomedical Science, Dankook University, Cheonan, Republic of Korea; Medical University of South Carolina, UNITED STATES

## Abstract

Understanding the biology behind the epidemicity and persistence of *Acinetobacter baumannii* in the hospital environment is critical to control outbreaks of infection. This study investigated the contributing factors to the epidemicity of carbapenem-resistant *A*. *baumannii* (CRAB) sequence type (ST) 191 by comparing the differences in biofilm formation, expression of biofilm-associated genes, and resistance to desiccation between major epidemic (*n* = 16), minor epidemic (*n* = 12), and sporadic (*n* = 12) clones. Biofilm mass was significantly greater in the major epidemic than the minor epidemic and sporadic clones. Major and minor epidemic clones expressed biofilm-associated genes, *abaI*, *bap*, *pgaABCD*, and *csuA/BABCDE*, higher than the sporadic clones in sessile conditions. The *csuC*, *csuD*, and *csuE* genes were more highly expressed in the major epidemic than minor epidemic clones. Interestingly, minor epidemic clones expressed more biofilm-associated genes than the major epidemic clone under planktonic conditions. Major epidemic clones were more resistant to desiccation than minor epidemic and sporadic clones on day 21. In conclusion, the epidemic CRAB ST191 clones exhibit a higher capacity to form biofilms, express the biofilm-associated genes under sessile conditions, and resist desiccation than sporadic clones. These phenotypic and genotypic characteristics of CRAB ST191 may account for the epidemicity of specific CRAB ST191 clones in the hospital.

## Introduction

*Acinetobacter baumannii* is a Gram-negative lactose non-fermenting pathogen capable of causing nosocomial infections among severely ill patients [1,2]. Once considered intrinsically tolerant to many antimicrobial agents, *A*. *baumannii* has become increasingly resistant to clinically useful antibiotics through the capacity to acquire antimicrobial resistance determinants. Furthermore, multi-drug resistant clones have emerged and circulated globally [3–5]. Resistance to myriad numbers of antimicrobial agents coupled with the ability of *A*. *baumannii* to persist and spread in healthcare settings has emerged as a global issue. Understanding the factors associated with the persistence and epidemicity of this opportunistic pathogen in healthcare environments may aid in the control of outbreaks in hospitals.

Multidrug efflux pumps have been suggested to influence colonization and persistence of *A*. *baumannii* [6]. Similarly, the *bla*_OXA-23_ and *bap* (*b*iofilm-*a*ssociated *p*rotein) genes are also possibly associated with the persistence of *A*. *baumannii* in hospitals [7]. Most studies have investigated the association of resistance to antibiotics with the circulation and/or persistence of *A*. *baumannii* isolates in healthcare systems. Espinal *et al*. [8] showed that biofilm formation increases the survival rate of *A*. *baumannii* on dry surfaces and may contribute to its persistence in the hospital environment, possibly leading to increased probability of *A*. *baumannii* causing nosocomial infections and hospital outbreaks. Biofilm formation in *A*. *baumannii* has also been linked to increased antimicrobial resistance, resistance to desiccation, and cleaning, and, therefore, represents an important factor associated with bacterial virulence [9–11]. Several genes are involved in various stages of *A*. *baumannii* biofilm formation, namely *ompA* encoding the major outer membrane protein OmpA [12], *bap* encoding the surface protein Bap [13], *csu* locus encoding the chaperone-usher pili assembly system [14], *abaI* encoding the quorum sensing auto-inducer [15], and *pga* locus encoding the proteins involved in the synthesis of cell-associated poly-β-(1–6)-N-acetyl glucosamine (PNAG) [16]. Inactivation of the *csuE* gene led to the total abolition of biofilm, whereas inactivation of other genes, such as *bap*, *abaI*, and *pgaABCD*, decreased the quantity or structure of biofilms [13–16].

We previously demonstrated that carbapenem-resistant *A*. *baumannii* (CRAB) sequence type (ST) 191 emerged as an epidemic clone and circulated in our hospital between 2011 and 2012 [17]. Of the CRAB isolates belonging to ST191, two pulsotypes differentiated by pulsed-field gel electrophoresis (PFGE) were predominantly appeared, whereas 23 pulsotypes included one or two isolates. To understand the epidemicity of specific pulsotypes of CRAB ST191 in the hospital, the present study investigated the biofilm formation, expression of biofilm-associated genes under sessile and planktonic conditions, and resistance to desiccation between epidemic and sporadic *A*. *baumannii* ST191 clones.

## Materials and Methods

### Bacterial isolates

Forty of 118 CRAB ST191 isolates between 2011 and 2012 were selected based on the pulsotypes in the previous study [17]. All CRAB ST191 isolates used in this study were extensively-drug resistant. Bacteria were cultured in lysogeny broth (LB) medium at 37°C.

### Selection of epidemic and sporadic *A*. *baumannii* ST191 clones

Epidemic and sporadic CRAB ST191 clones were defined based on the pulsotypes generated by PFGE of CRAB isolates in the previous study [17]. Two predominant pulsotypes of arbitrary pulsotypes 5 (*n* = 20) and 18 (*n* = 17) were considered the major epidemic clones. For major epidemic clones, 16 isolates (eight isolates from each pulsotype) that showed different PFGE patterns in the same pulsotypes were selected. Minor epidemic clones were defined as pulsotypes containing six to seven isolates. Twelve isolates (six from each pulsotype) from arbitrary pulsotypes 12 (*n* = 7) and 20 (*n* = 6), falling within the scope of the definition of minor epidemic clones were selected for the study. Sporadic clones were defined as pulsotypes containing only one isolate and 12 isolates from each pulsotype were selected for the sporadic clones in the present study.

### Biofilm formation assay

The biofilm mass produced by *A*. *baumannii* isolates was measured using a biofilm formation assay in 5 ml polystyrene tubes, based on procedures outlined by Heilman *et al*. [18] with some modifications. Briefly, overnight bacterial cultures were adjusted to a turbidity of 2.0 at 600 nm using Mueller-Hinton (MH) broth and diluted 200-fold in MH medium. Aliquots of 1 ml bacterial suspension were inoculated into 5 ml polystyrene tubes and incubated for 24 h at 30°C without shaking. The planktonic or loosely attached cells were removed and tubes were then washed twice with 1 ml phosphate-buffered saline (PBS). The tubes were air-dried and stained with crystal violet (0.1% v⁄v) for 15 min. The stained biofilms were then solubilized with 1 ml ethanol for 5 min, and 200 μl of samples were transferred into a 96-well plate and the turbidity was measured at 570 nm using a microplate reader (Molecular Devices, Sunnyvale, CA). In addition, the turbidity was also measured at 600 nm prior to staining of biofilm mass to compensate for growth differences in the isolates. Each experiment was performed in triplicate and repeated three times.

### RNA isolation and reverse transcriptase-quantitative PCR (RT-qPCR)

Gene expression associated with biofilm formation was analyzed in both planktonic and sessile conditions using a previously described method [19] with some modifications. In brief, overnight bacterial culture was adjusted to OD_600_ 2.0 and diluted 200-fold in MH broth. A 1 ml aliquot of bacterial suspension was inoculated into a 5 ml polystyrene tube and incubated with shaking for planktonic condition and without shaking for sessile condition for 24 h. All bacterial cells incubated with shaking were collected for RNA extraction. Non-adherent cells from without shaking cultures were aspirated and cells attached to polystyrene tubes were scraped off and used for RNA extraction. Total RNA was extracted using an RNeasy Mini Kit (Qiagen, Valencia CA) according to the manufacturer’s instructions. cDNA was generated by the reverse transcription of 2 μg of total RNA using oligo dT primers and RevertAid reverse transcriptase in a total reaction volume of 20 μl (Thermo Scientific, Lithuania). Forward and reverse primers for each gene were designed using Primer Express Software 3.0 and they were listed in Table 1. Primers specific for the *bap* and 16S rRNA genes described in the previous studies were used [20,21]. RT-qPCR was performed as previously described [19]. Quantification of gene transcripts was performed using the Power SYBR Green PCR Master Mix (Applied Biosystems, Waltham, MA) on a StepOnePlus Real-Time PCR System (Applied Biosystems). *A*. *baumannii* ATCC 19606^T^ was used as the reference strain, because the ability of this strain to form biofilm mass was higher than clinical *A*. *baumannii* isolates [22] and *A*. *baumannii* ATCC 19606^T^ carried all tested biofilm-associated genes. Fold changes in gene expression were calculated using the comparative C*t* method (2^-ΔΔC^_T_) [23] and samples were normalized to 16S rRNA expression. Each experiment was performed in triplicate and repeated two times.

**Table 1:** Oligonucleotide primers used in this study.

Gene	Primer sequences (5'➔3')	References
*abaI*	CCGCCTTCCTCTAGCAGTCA	This study
	AAAACCCGCAGCACGTAATAA	
*pgaA*	GCCGACGGTCGCGATAC	This study
	ATGCACATCACCAAAACGGTACT	
*pgaC*	GCGTATCCGTACCCGTTCAA	This study
	TGAACAGCGCTCTTACGAAAAG	
*csuC*	AAAGCAGGCGAGAAGCATATG	This study
	GGATCGGCAACTCATCTACAATC	
*csuD*	ACCCTATCAAGGCGGTTCAAC	This study
	CGACGATAGCCGTCATTATCTACA	
*csuE*	TCAGACCGGAGAAAAACTTAACG	This study
	GCCGGAAGCCGTATGTAGAA	
*bap*	AATGCACCGGTACTTGATCC	[20]
	TATTGCCTGCAGGGTCAGTT	
16S rRNA	ACTCCTACGGGAGGCAGCAGT	[21]
	TATTACCGCGGCTGCTGGC	

### Bacterial survival under desiccation

A total of 15 isolates, five from each group (i.e., highest, moderate, and low biofilm-forming isolates from major epidemic, minor epidemic, and sporadic clones, respectively), were selected and their resistance to desiccation was determined over a period of 21 days using the method described by Jawad *et al*. [24] with some modifications. A 1 ml aliquot of overnight LB culture was centrifuged for 5 min at 11,600 × *g*. Cells were then washed once with 1 ml distilled water and resuspended in 1 ml distilled water. Aliquots of 20 μl each were deposited into 1.5 ml sterilized tubes. The tubes were opened, placed in a rack, and kept in airtight transparent plastic boxes. A saturated solution of CaCl_2_·6H_2_O was used to maintain constant humidity at 31% ± 3%. A digital thermohygrometer (Dretec, Japan) was used to monitor both humidity and temperature. Viable cells were determined on days 0, 2, 5, 9, 11, 15, and 21 as previously described [24].

### Statistical analysis

Data were analyzed using R 3.3.1 (https://www.r-project.org/) and FSA package version 0.8.8 (https://github.com/droglenc/FSA). The non-parametric Kruskal-Wallis test was used to calculate significant difference between three clones. If statistical significance was observed, *P*-value was calculated by Dunn’s multiple comparison method and then adjusted for Bonferroni method. Spearman’s correlations test was used to determine the association between biofilm formation and bacterial survival under desiccation. Differences of *P* <0.05 were considered as statistically significant.

## Results

### Biofilm quantification

**Fig 1:**
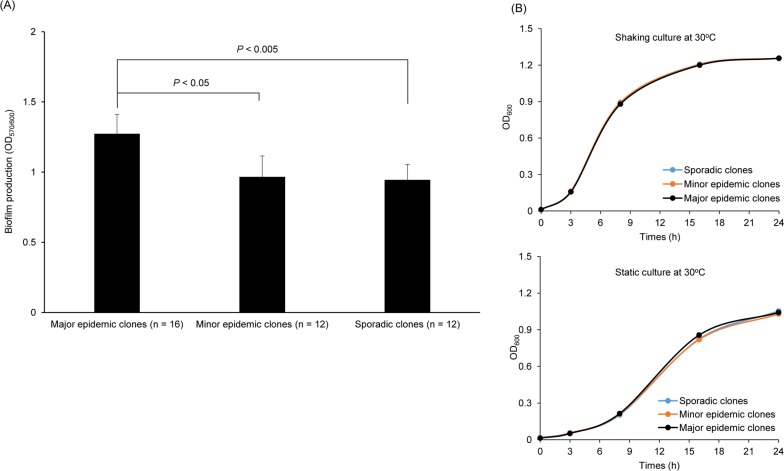
Biofilm formation and growth of 40 carbapenem-resistant *A*. *baumannii* ST191 isolates. (A) Bacteria were cultured in 5 ml polystyrene tubes for 24 h at 30°C. Biofilm formation was quantified by calculating the ratio of OD_570_/OD_600_. Data are presented as the mean ± standard deviation of three independent experiments, each performed in triplicate. (B) Bacteria were cultured in 5 ml polystyrene tubes for 24 h at 30°C with or without shaking. Optical density at 600 nm was determined in each time point. Data are presented as the mean value of two independent experiments.

Based on the pulsotypes of the CRAB isolates from a single hospital between January 2011 and August 2012 [17], a total of 40 isolates [major epidemic (*n* = 16), minor epidemic (*n* = 12), and sporadic (*n* = 12) clones] were selected among the 118 CRAB ST191 isolates. The ability of three different CRAB clones to form biofilms was determined. Major epidemic clones (1.27 ± 0.14) formed significantly more biofilm mass than the minor epidemic (0.97 ± 0.15) and sporadic (0.94 ± 0.11) clones (Fig 1A). However, no significant difference in biofilm mass was observed between the minor epidemic and sporadic clones. There was no significant difference in bacterial growth at 30°C under shaking or static culture between three different clones (Fig 1B).

### Biofilm-associated gene expression under sessile conditions

**Fig 2:**
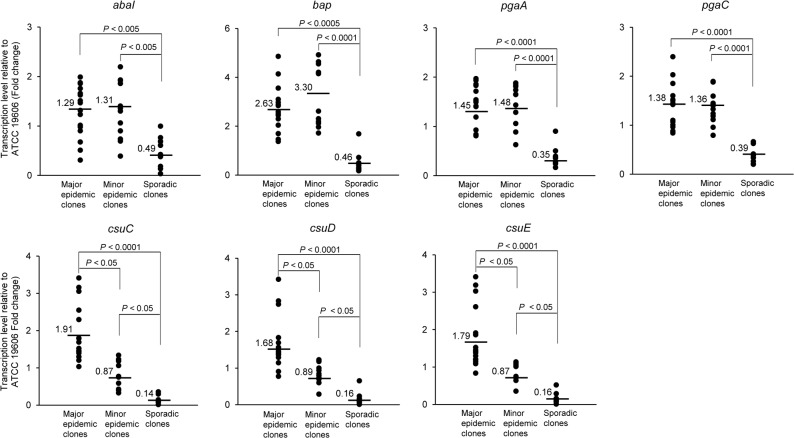
Expression of biofilm-associated genes in carbapenem-resistant *A*. *baumannii* ST191 isolates in sessile conditions. Bacteria were cultured in 5 ml polystyrene tubes for 24 h at 30°C without shaking. Non-adherent cells were removed and cells attached to polystyrene tubes were collected for RNA extraction. Transcriptional levels of the *abaI*, *bap*, *pgaA*, *pgaC*, *csuC*, *csuD*, and *csuE* genes in carbapenem-resistant *A*. *baumannii* isolates were determined using RT-qPCR. The dots in the figure are the mean of expression levels of genes in each isolate relative to expression of genes in *A*. *baumannii* ATCC 19606^T^ and the values indicates the mean of gene expression for the entire group. Data were obtained from two independent experiments, each performed in triplicate.

We determined the presence of biofilm-associated genes, *bap*, *abaI*, *pgaABCD*, and *csuA/BABCDE*, in the 40 CRAB ST191 isolates using PCR analysis. This revealed that all isolates carried the biofilm-associated genes tested (data not shown). Next, the expression levels of biofilm-associated genes were determined using RT-qPCR. Since the *pgaABCD* and *csuA/BABCDE* are operons and their cluster of genes are under the control of single promoter [14,15,25], the *pgaA* and *pgaC* genes were selected as a representative of the *pgaABCD* operon, and the *csuC*, *csuD*, and *csuE* genes were selected to represent the *csuA/BABCDE* operon. Compared to sporadic clones, the major epidemic and minor epidemic clones expressed higher levels of *abaI*, *bap*, *pgaAC*, and *csuCDE* genes in sessile conditions (Fig 2, S1 Table). No significant differences in the expression of *abaI*, *bap*, *pgaA*, and *pgaC* were observed between the major epidemic and minor epidemic clones (Fig 2). However, expression of *csuC*, *csuD*, and *csuE* was significantly different between the major epidemic and minor epidemic clones (Fig 2).

### Biofilm-associated gene expression under planktonic conditions

**Fig 3:**
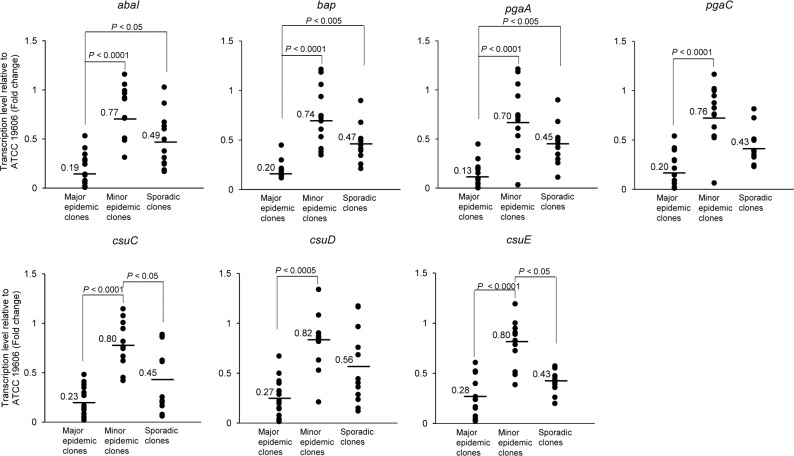
Expression of biofilm-associated genes in carbapenem-resistant *A*. *baumannii* ST191 isolates in planktonic conditions. Bacteria were cultured in polystyrene tubes for 24 h at 30°C with shaking. All bacterial cells were collected for RNA extraction. Transcriptional levels of the *abaI*, *bap*, *pgaA*, *pgaC*, *csuC*, *csuD*, and *csuE* genes in carbapenem-resistant *A*. *baumannii* isolates were determined using RT-qPCR. The dots represent the mean of expression levels of genes in each isolate relative to expression of genes in *A*. *baumannii* ATCC 19606^T^ and the values indicates the mean of gene expression for the entire group. Data were obtained from two independent experiments, each performed in triplicate.

We further explored the expression levels of biofilm-associated genes in CRAB ST191 isolates under planktonic conditions. Contrary to the results observed in sessile conditions, expression of *abaI*, *bap*, *pgaA*, and *csuE* in the major epidemic clones was significantly lower than that of the minor epidemic and sporadic clones (Fig 3, S2 Table). Furthermore, the minor epidemic clones expressed higher levels of *abaI*, *bap*, *pgaA*, *pgaC*, *csuC*, *csuD*, and *cusE* than the major epidemic clones (Fig 3). Similarly, the sporadic clones also expressed significant levels of *abaI*, *bap*, and *pgaA* than the major epidemic clones.

### Resistance to desiccation

**Fig 4:**
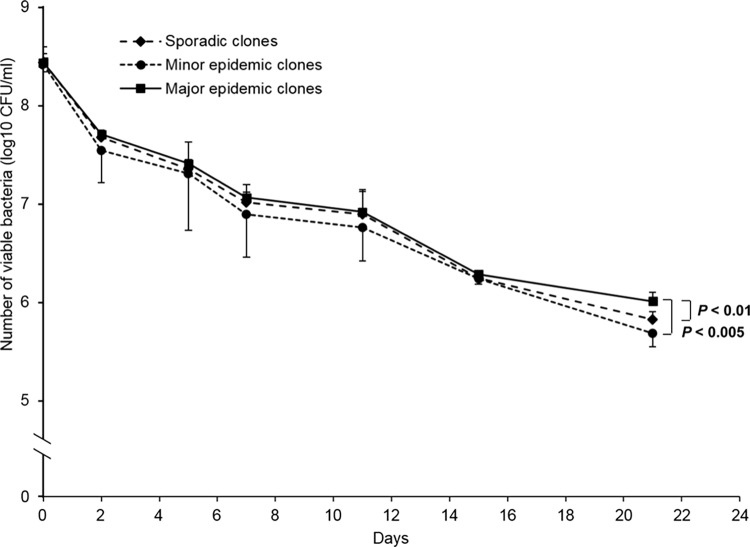
Resistance to desiccation of carbapenem-resistant *A*. *baumannii* isolates. A total of 15 isolates, five each from major epidemic, minor epidemic, and sporadic clones, were selected and subjected to desiccation assay. Bacteria were inoculated into a 1.5 ml sterilized microcentrifugation tube and incubated at room temperature with a relative humidity of 31% for 21 days. Approximately ~2.7 x 10^8^ CFUs were used as starting bacterial inoculum. Data are presented as the mean values of two independent experiments of each group.

Fifteen isolates were subjected to a survival assay under desiccation conditions for 21 days. Approximately 2.7 x 10^8^ CFUs were inoculated at day 0 and viable bacteria were counted on days 2, 5, 9, 11, 15, and 21. No significant difference in bacterial viability was observed on days 2, 5, 7, 11, and 15 between all the three groups (Fig 4). However, the number of viable cells belonging to the major epidemic clones (1.08 x 10^6^ CFUs) was significantly higher than that of the minor epidemic (5.21 x 10^5^ CFUs)) and sporadic (6.86 x 10^5^ CFUs) clones on day 21. No significant difference was observed between the minor epidemic and sporadic clones on the same day. To determine whether biofilm formation of each bacterium was correlated with resistance to desiccation, Spearman’s correlation test was performed. There was a weak positive correlation between biofilm formation and resistance to desiccation (Spearman’s correlation coefficient, 0.33).

## Discussion

*A*. *baumannii* has the potential to colonize and persist in hospital environments and on medical devices [9,26], possibly due to its ability to form biofilms and resistance to antimicrobial agents [10]. The overall data of the current study agreed with the previous findings that *A*. *baumannii* has an enormous capacity to form biofilms and that the epidemic clones have higher biofilm-forming ability than sporadic clones. The ability to form high biofilms is an effective strategy for microbes to boost their survival in harsh and stressed conditions [10]. Examples of such conditions include desiccation, cleaning, and antibiotic exposure. In addition, biofilm communities are excellent milieu for horizontal gene transfer and may lead to the acquisition of resistant genes [27]. Therefore, harsh conditions propagate the selection of resistant and higher biofilm-forming strains within biofilm communities. Acquisition of these properties has made *A*. *baumannii* able to survive on surfaces for several days, and resist desiccation and disinfection leading to multiple outbreaks in the hospital [28,29].

An interesting finding of the current study was that the major epidemic clones of CRAB ST191 showed a significantly higher capacity to form biofilms than both the minor epidemic and sporadic clones. This phenotypic phenomenon is highly correlated with the expression of the *csuC*, *csuD*, and *csuE* genes in sessile culture conditions. Furthermore, a high expression of all tested biofilm-associated genes was observed in the major epidemic clones of CRAB ST191 compared to that of the sporadic clones. Although there was no significant difference in biofilm formation between the minor epidemic and sporadic clones of CRAB ST191, expression levels of all tested biofilm-associated genes under sessile conditions were significantly higher in minor epidemic clones that those in sporadic clones. Pilus production mediated by the CsuA/BABCDE usher-chaperone assembly system is required for the initial step of bacterial attachment on the abiotic surfaces, followed by the full development of biofilms [14]. Tomaras *et al*. [14] demonstrated that this pilus is critical for adherence to and biofilm formation of *A*. *baumannii* ATCC 19606^T^ on abiotic surfaces. Inactivation of the *csu*E gene results in the abolition of pili production as well as cell attachment and biofilm formation [14]. Therefore, an appropriate expression of this appendage in *A*. *baumannii* under sessile conditions leads to the strong attachment of bacteria to solid surfaces and medical devices, and may be a determining factor in epidemicity of *A*. *baumannii*. Strikingly, under planktonic conditions, both the minor epidemic and sporadic ST191 clones expressed higher levels of all biofilm-associated genes than the major epidemic ST191 clones. These results suggest that major epidemic ST191 clones may tightly regulate expression of biofilm-associated genes under different culture conditions. Even though no difference in biofilm formation was observed between the minor epidemic and sporadic clones in the present study, the minor epidemic clones expressed higher levels of all biofilm-associated genes under planktonic conditions. The possible explanation for this discrepancy is that other factors such as two-component system AdeRS [30], LH092_11085 gene products [31], and many over-expressed or specifically expressed gene products in sessile bacteria [32] may be involved in biofilm formation of CRAB ST191.

Major epidemic ST191 clones showed an ability to resist desiccation on dry surfaces for long periods, which is consistent with the overall capacity of *A*. *baumannii* to resist drought [8,24,33]. No differences in viable bacterial counts were observed between the three groups for 15 days. However, on day 21, the number of viable bacterial cells was higher in the major epidemic clones than the minor epidemic and sporadic clones of CRAB ST191. Viable counts between the minor epidemic and sporadic clones on the same day were not significantly different. Major epidemic ST191 clones are more resistant to desiccation than minor epidemic and sporadic ST191 clones. Similar observations were reported by Espinal *et al*. [8], although the present study did not explore the overall survival times of all isolates. However, Spearman’s correlation test showed a weak positive correlation between biofilm formation and resistance to desiccation, although major epidemic clones produced significantly more biofilm mass than the minor epidemic and sporadic clones.

In summary, the present study proved that high biofilm formation and expression of pili usher operon *csuA/BABCDE* under sessile conditions are associated with the specific pulsotypes of CRAB ST191. In addition, major epidemic ST191 clones are more resistant to desiccation than minor epidemic and sporadic ST191 clones. Together, these phenotypic and genotypic characteristics of CRAB ST191 may be responsible for the persistence and prevalence of specific CRAB ST191 pulsotypes in the study hospital. We tested one specific genotype of *A*. *baumannii* ST191, the most prevalent ST in Korea, in this study. Therefore, it should be determined whether our results obtained from the CRAB ST191 can be applied to other epidemic or international clones of *A*. *baumannii*.

## Supporting Information

S1 Table Expression of biofilm-associated genes of *Acinetobacter baumannii* isolates between three different clones under sessile conditions. (XLSX)

S2 Table Expression of biofilm-associated genes of *Acinetobacter baumannii* isolates between three different clones under planktonic conditions. (XLSX)
